# Effect of Composite-Region Fraction and TiCp Content on the Mechanical Properties of H13 Steel Matrix Composites with Honeycomb Architecture

**DOI:** 10.3390/ma18051128

**Published:** 2025-03-02

**Authors:** Shengpeng Li, Dehong Lu, Jiaxing Zhong, Tao He, Yehua Jiang

**Affiliations:** 1 Faculty of Materials Science and Engineering, Kunming University of Science and Technology, Kunming 650093, China; 19909459125@163.com (S.L.); 13330496952@163.com (J.Z.); hetao231320@163.com (T.H.); 2 National and Local Joint Engineering Research Center for Advanced Solidification Forming and Equipment Technology of Metals, Kunming 650093, China; jiangyehua@kmust.edu.cn

**Keywords:** honeycomb-architecture composites, composite region fraction, TiCp content, H13 steel, mechanical properties, failure analysis

## Abstract

In order to improve the strength and toughness-matching of metal matrix composites and enhance the mechanical properties of ceramic-reinforced iron matrix composites with a honeycomb architecture, TiCp/H13 steel composites with a honeycomb architecture were successfully prepared using squeeze-infiltration technology, in which the composite region was the honeycomb wall and the steel matrix was the honeycomb core. The effects of the composite-region fraction and TiCp content in the composite region on the compressive mechanical properties of the composites were studied, and the fracture mode and cracking behavior were analyzed. The results show that TiCp was evenly distributed in the composites region, and the interface of TiCp/H13 steel was tightly bonded without obvious defects. With the same TiCp content, the compressive strength of honeycomb-architecture composites first increased and then decreased with the increase in the composite-region volume fraction, and the highest strength was obtained at 50 vol.% of the composite region. The influence factor of the composite-region volume fraction on the strength was −38.3 MPa/%. Meanwhile, the fracture strain of the architecture composites decreased gradually. The influence factor of the composite-region volume fraction on plasticity was −0.25%/%. With the same composite-region fraction, both the compressive strength and plasticity of the composite decreased gradually with the increase in TiCp content (35 vol.%, 50 vol.%, and 65 vol.%). The influence factor of TiCp content on the strength was −21.4 MPa/%, and its influence factor on plasticity was −0.34%/%. The maximum compressive strength (2288.1 MPa) was obtained in the architecture composite with 50 vol.% of the composite region and 35 vol.% of TiCp, and the highest plasticity (25.9%) was obtained for the architecture composite, with 35 vol.% of the composite region and 35 vol.% of TiCp. Compared to those of common ZTA/iron honeycomb-architecture composites, the comprehensive mechanical properties of the TiCp/H13 steel matrix honeycomb-architecture composites were greatly improved. It showed good energy-absorption characteristics during compression.

## 1. Introduction

Steel and iron-based wear-resistant materials [1] are widely used in engineering machinery for mining [2], mineral processing [3], and the cement industry [4,5]. Traditional wear-resistant steel and iron [6] encounter limitations in the face of complex high-pressure wear conditions, and it is difficult to meet the requirements of high strength, high toughness, and high wear at the same time [7,8]. For example, when mineral materials are crushed by a roller press, a high-chromium cast-iron roll with high hardness easily flakes off from the surface because of brittleness, resulting in serious wear and shortened service life [9,10,11]. In order to improve wear resistance, a kind of composite roll embedded with cemented carbide studs on the wear surface has been developed and successfully applied in a large number of applications [10]. However, the manufacturing of composite rolls requires a large amount of hole machining and the manual inlaying of studs, along with a long cycle and high cost. At the same time, the interface bonding between the cemented carbide studs and the steel matrix is low, and the studs easily fall off during service. These abnormal losses prevent the roller press from making use of the high hardness and wear resistance of cemented carbide studs [12].

For conventional composites containing uniformly distributed ceramic particles, composite hardness generally increases with the increase in ceramic reinforcements, but plasticity and toughness significantly decrease, so it is impossible to obtain high hardness and superior toughness at the same time, which is detrimental to the wear resistance of the composites and limits their application as high-performance wear-resistant materials. In recent years, architecture composites, as a new kind of material with a unique architecture, high strength, high toughness, and high wear resistance, have shown great potential in the field of modern engineering [13,14,15,16]. For example, Liu et al. [17] studied the influence of structural parameters on the bending fracture behavior of laminated composite Ti-(TiBw/Ti). The results show that with a decrease in the Ti layer’s thickness and an increase in the TiBw volume fraction, the alloy undergoes a tough–brittle transition, and a laminated composite with a weak interface exhibits excellent fracture toughness under notch-crack suppression orientation. Wan et al. [18] prepared a layered high-entropy alloy (HEA) granular-reinforced Al matrix composite. The results show that the composite with an appropriate proportion (19 vol.%) of layered architecture obtained a good combination of yield strength (445.2 MPa) and fracture strain (5.4%). This can be attributed to the strengthening and toughening effects induced by the bimodal gain architecture. The strengthening of the intermediate architecture of the composite mainly comes from the strengthening of the grain boundary. The balance of strength and toughness can be achieved by reasonably adjusting the material combination and thickness distribution between the layers [19]. The network architecture, with its regular spatial architecture, shows excellent compressive mechanical properties and energy-absorption characteristics [20,21,22]. Zhong et al. [23] successfully prepared a mesh polyphase (TiB + TiC + Ti_3_Si)/TC4 composite with a bending strength of up to 1863 MPa and a toughness (K_Ic_) of 17.8 MPa·m^1/2^. The strengthening of the mesh composite was attributed to the collaborative load transfer of the mixed reinforcement material. The honeycomb architecture, with its unique porous architecture, can effectively disperse the load, improve the compressive and bending properties, and absorb energy through deformation when impacted, protecting the internal structure. It is widely used in many fields to improve the comprehensive performance of materials [24,25,26]. Zhang et al. [27] prepared a honeycomb-architecture zirconia-toughened alumina particle (ZTAp)/high-chromium cast-iron (HCCI) composite using the pressureless infiltration casting process. With an increase in the pore size, the compressive mechanical properties were gradually improved, and the highest compressive strength was 1286.14 ± 15.97 MPa when the pore size was 12 mm. Therefore, such a typical architecture design can significantly improve the strength and toughness-matching of metal matrix composites. Honeycomb-architecture ZTAp/HCCI composites have been widely used in various wear-resistant products, such as vertical grinding rolls and plate hammers [28]. Nevertheless, ceramic-reinforced iron matrix composites with honeycomb architecture still have significant potential for improvement in terms of mechanical properties. Firstly, the reinforcement ZTA particles are typically too coarse (usually more than 1 mm), making them prone to cracking [29]. Secondly, although high-chromium cast iron has high strength, it is brittle and prone to cracking. Additionally, the poor wettability between ZTA and steel matrix leads to a weak ZTA/steel interface, making it easy to crack [27].

Titanium carbide particle (TiCp)-reinforced steel matrix composites combine the excellent ductility of steel with the high hardness of ceramics, resulting in superior comprehensive mechanical properties and unique performance characteristics, such as high specific elastic modulus and strength, outstanding high-temperature stability, and enhanced wear resistance. Consequently, these composites are widely used as mold materials and wear-resistant materials [30,31,32,33].

In this study, TiCp and H13 steel were used as composite systems to replace ZTA and high-chromium cast iron for fabricating honeycomb-architecture composites. This approach improves the strength and toughness-matching of metal matrix composites, significantly enhancing the comprehensive mechanical properties of the honeycomb iron matrix composites. Specifically, TiCp with a size of 10 μm was used as the reinforcement, replacing the millimeter-sized ZTAp to reduce the probability of ceramic particle cracking and to improve the wettability of the ceramic with the steel matrix; H13 steel was selected as the matrix to enhance the comprehensive properties of strength and toughness, thereby improving crack resistance. The mechanical properties of the architecture composite, especially the effects of composite-region volume fraction and TiCp content, were studied, and the failure modes and cracking behavior of the composite were analyzed.

## 2. Materials and Methods

### 2.1. Raw Materials

**Figure 1:**
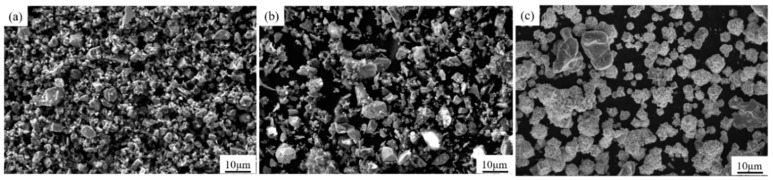
SEM micrographs of raw material particles: (**a**) TiC; (**b**) Cr-Fe; (**c**) Ni-Fe.

TiC particles were used as the reinforcement, with a purity of 99.99%, hardness of 2400–2800 HV, and a particle size of 5~10 μm, produced by the MCC New Materials Company (Tangshan, China), as shown in Figure 1a. Cr-Fe powder was used to adjust the volume fraction of TiCp in the composite, with a purity of 99.99%, particle size of 5~10 μm, and hardness of 300–400 HV, which was produced by the Hangzhou Metal Materials Company (Xingtai, Hebei, China), as shown in Figure 1b. Ni-Fe powder was added as an activated powder to improve the mechanical properties of the composite [34], with a purity of 99.99%, hardness of 115–140 HV, and a particle size of 5~10 μm, produced by the Hebei Enyi Metal Materials Company (Qinghe, Handan, China), as shown in Figure 1c.

4Cr5MoSiV1 steel (H13 steel) [35] was selected as the matrix, with its chemical composition shown in Table 1. These data were obtained by chemical analysis. This steel is known for its excellent heat resistance, wear resistance, and impact resistance, making it widely used in high-temperature, high-pressure, and severe wear conditions.

**Figure 2:**
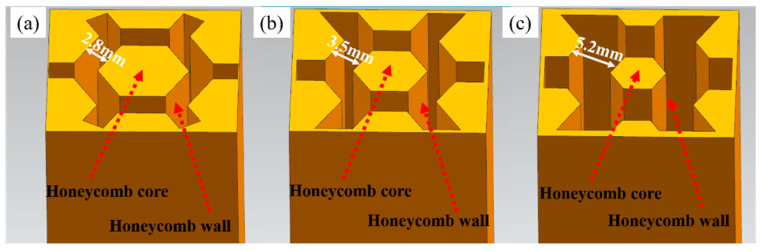
Honeycomb mold diagrams for different volume fractions of the composite region: (**a**) 35 vol.%; (**b**) 50 vol.%; (**c**) 65 vol.%.

The honeycomb architecture of the composites is shown in Figure 2, with the parameters provided in Table 2. The honeycomb wall represents the composite region, while the honeycomb core constitutes the matrix region. The average size of each sample is 20 mm × 20 mm × 30 mm. As the honeycomb wall thickness increases from 2.8 mm to 3.5 mm and 5.2 mm, the volume fraction of the composite region increases from 35 vol.% to 50 vol.% and 65 vol.%, respectively.

In addition to the changes in the volume fraction of the composite region, the volume fraction of TiCp within the composite region was also adjusted. Nine samples were prepared. The specific volume fraction of the composite region and TiCp content for each composite are provided in Table 3.

### 2.2. Fabrication of the Architecture Composites

**Figure 3:**
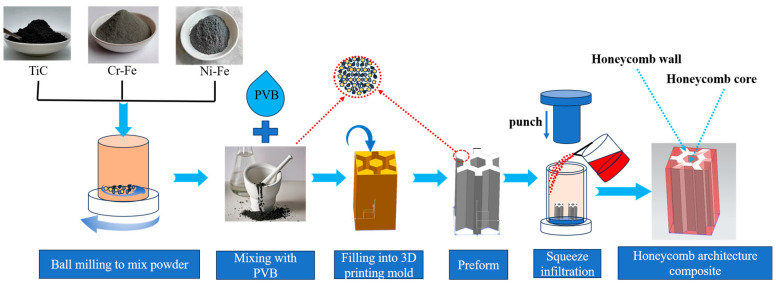
Schematic of fabrication of the architecture composite.

Figure 3 shows a schematic diagram of the fabrication process for the architecture composites. The TiCp architecture preforms were prepared as follows: First, the raw materials, namely TiC particles, Cr-Fe powder, and Ni-Fe powder, were weighed according to the volume fraction of the composite region and TiCp content, then put into a ball mill for dry mixing. Agate balls were used as the grinding medium, with three sizes of balls, specifically 4 mm, 2 mm, and 1 mm diameter. The speed of the ball mill was set to 150 r/min, and the ball-grinding time was 8 h, with a ball-to-material ratio of 5:1. Next, the mixed powder was blended evenly with a polyvinyl butyric aldehyde (PVB) binder at a ratio of 8:1, and filled into a 3D-printed plastic honeycomb mold. The mixture was then pressed under a pressure of 1 MPa to fabricate the TiCp honeycomb preform. Finally, the preform was sintered in a vacuum furnace at 1300 °C to decompose and vaporize the binder and obtain a certain strength. During the sintering process, the sample experienced a shrinkage of 1%.

H13 steel was melted to 1600 °C using a medium-frequency induction furnace, and the TiCp preform was fixed in a cylindrical steel mold. Then, the molten steel at a temperature of 1580 ± 10 °C was poured into the mold and pressed under a pressure of 50 MPa to infiltrate the preform. Finally, the honeycomb-architecture composite was obtained after solidification.

The H13 steel matrix was also fabricated using the same squeeze-casting process under the same conditions.

### 2.3. Heat Treatment of the Composites

**Figure 4:**
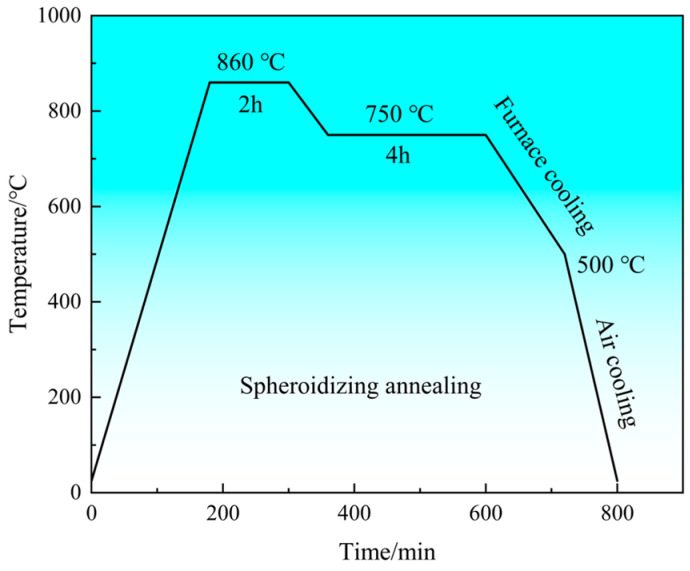
Spheroidizing annealing process.

H13 steel and the composites were treated with spheroidizing annealing to make the microstructure uniform, reduce hardness, eliminate mesh carbide, improve the shape of the carbides, and prepare the microstructure for the final heat treatment. The specific process is shown in Figure 4. During this process, the atmospheric environment was air.

**Figure 5:**
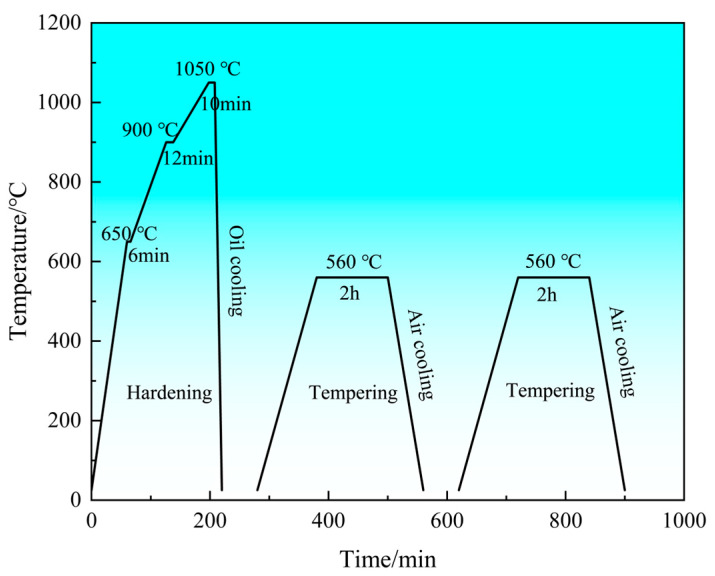
Quenching and high-temperature tempering process of materials.

The final heat treatment of the material comprised quenching and twice high-temperature tempering. The specific process is shown in Figure 5.

### 2.4. Material Performance Tests

#### 2.4.1. Hardness Testing

The microhardness of the H13 steel matrix and the composite region of the composites was measured using an HVS-1000AT automatic microhardness tester (HVS-1000AT, Laizhou Hengyi Test Instrument Company Ltd., Yantai, China). To ensure the accuracy of the data, five points were selected on each sample for measurement, and the average value was taken as the final result. The samples were tested according to the metal material-hardness testing standard. During the microhardness test, a load of 0.98 N was applied, and the loading time was 10 s.

#### 2.4.2. Compression Performance Testing

**Figure 6:**
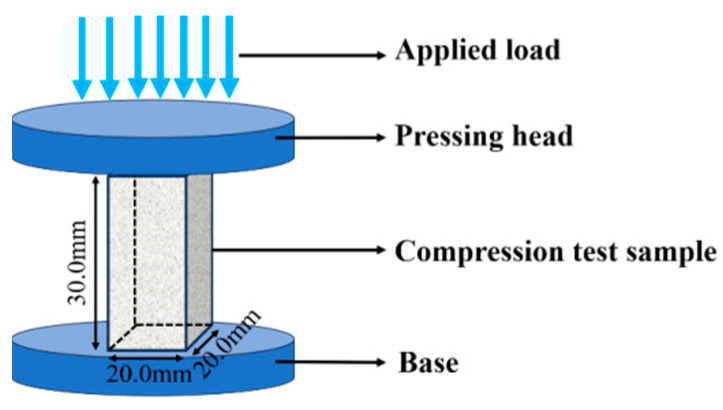
Schematic diagram of compression testing.

The sample for the compression performance test was obtained by wire cutting and polishing, with dimensions of 20 mm × 20 mm × 30 mm. As a wear-resistant material, the sample is primarily subjected to compression and shear. Therefore, compressive performance was used to evaluate the mechanical properties of the material in this study. The compression performance test was carried out using a PWS-1000 electrohydraulic mechanical testing machine (manufactured by the Beijing Institute of Photonics Science and Technology), as shown in Figure 6. The machine has a maximum load capacity of 1000 kN, and the loading rate is 0.65 mm/min. Two samples of each material were tested, and the average value was taken.

### 2.5. Material Characterization Methods

The microstructure and compressive fracture morphology of the materials were observed using a scanning electron microscope (SEM5000; LEO-1450, SEMTech Solutions, North Billerica, MA, USA). Element distribution in both the matrix region and composite region was detected with electron probe microanalysis (EPMA; JXA-iHP200F, JEOL, Frenchs Forest, NSW, Australia JXA-IHP 200F). The volume fraction of TiCp in the composite region was calculated using Image J software https://imagej.net/ij/ (Fiji) from five SEM images of the same sample, with the average value taken. The phases of the composites were detected using a Dutch Panak Pyrean sharp X-ray diffractometer (XRD) at a scanning speed of 2°/min. Additionally, a Phoenix v|tome|x X-ray computed tomography (CT; Baker Hughes, Germany) machine was used to detect crack distribution inside the composites after the compression tests, and Avizo (2019.1) software was used for three-dimensional reconstruction.

## 3. Results

### 3.1. Phase of the Materials

**Figure 7:**
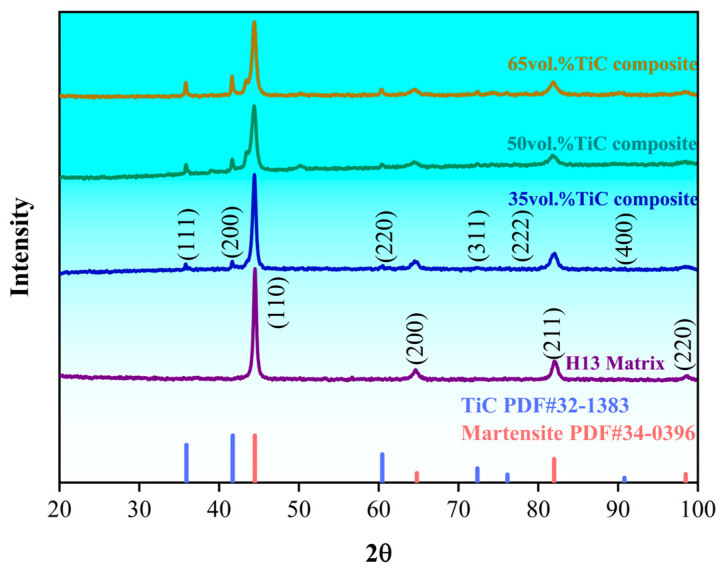
XRD patterns of different materials.

The XRD patterns of the prepared H13 steel matrix and the honeycomb composites are shown in Figure 7. The microstructure of the H13 steel matrix consists of a single martensitic phase without an obvious austenite peak. The composites primarily contain two phases: martensite and TiC. In the 35 vol.% TiCp composite, the intensity of the martensite diffraction peak is relatively high due to the low amount of TiC. As the TiCp content increases, the peak intensity of TiC also increases, while the martensite peak intensity gradually decreases.

### 3.2. Microstructure of Composites with Honeycomb Architecture

**Figure 8:**
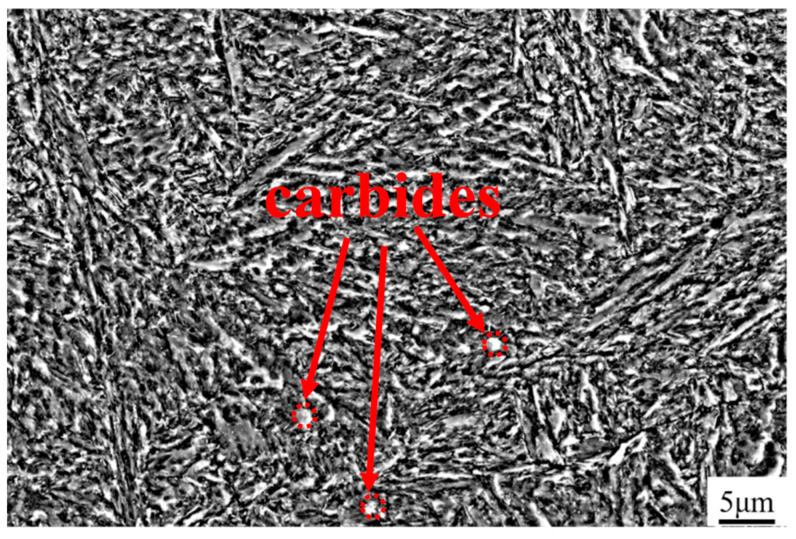
Microstructure of H13 steel matrix after heat treatment.

Figure 8 shows the microstructure of the H13 steel matrix after heat treatment. It primarily consists of tempered martensite and a small amount of carbide, while the carbide at the grain boundary as cast is basically eliminated. The tempered martensite exhibits a bamboo leaf-shaped structure with fine and small laths, which contributes to the improved strength and wear resistance of the material [36].

**Figure 9:**
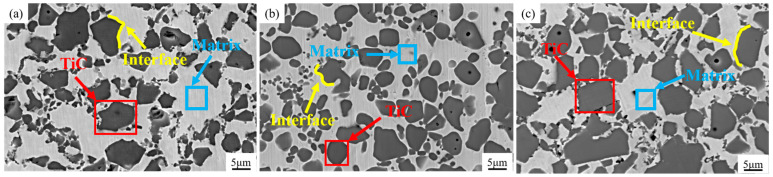
Microstructure of composites with different TiCp content: (**a**) 35 vol.%; (**b**) 50 vol.%; (**c**) 65 vol.%.

Figure 9 shows the microstructure of TiCp/H13 steel architecture composites with different TiCp content, with Figure 9a–c containing 35 vol.%, 50 vol.%, and 65 vol.% of TiCp, respectively. All composites are primarily composed of black TiCp and light gray matrix, without obvious TiCp agglomeration. The interface of TiCp/H13 steel is tight, with no defects such as shrinkage holes and cracks. According to quantitative metallographic analysis, the actual volume fractions of TiCp in the three honeycomb composites (volume fraction of 35%, 50%, and 65%) are 36.1%, 51.0%, and 61.9%, respectively, which are close to the design values.

**Figure 10:**
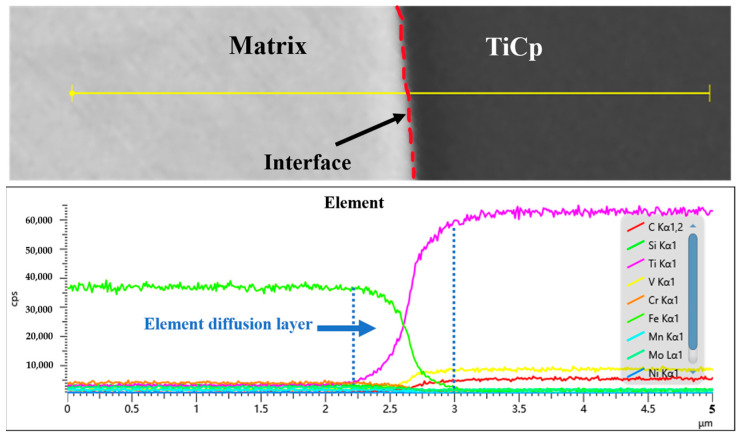
Elemental distribution at TiC/H13 interface.

EDS was used to characterize the element distribution at the TiCp/steel interface in the 35 vol.% TiCp composite, as shown in Figure 10. At the TiCp/steel interface, Ti and C in TiCp diffuse into the steel, and Fe from the matrix diffuses into the TiC. Vanadium also diffuses from the steel matrix to the TiCp, forming an interfacial transition layer with a thickness of 0.7 μm, which should be beneficial to the interfacial bonding.

**Figure 11:**
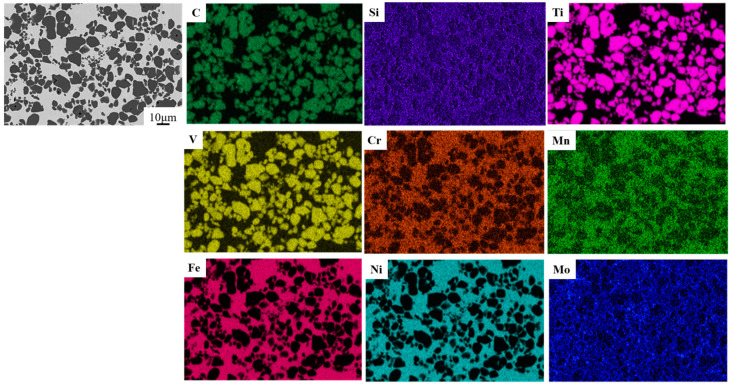
SEM image and element distribution in TiCp/H13 composite region.

The distribution of elements in the composite region of the architecture composites was further characterized, as shown in Figure 11. Cr, Mo, Si, and Mn are primarily distributed in the H13 steel matrix, while Ni is uniformly dissolved in the matrix, and V is solidly dissolved in TiCp to form (Ti,V)C. This is because TiC and VC have a face-centered cubic (FCC) structure and similar lattice parameters (0.4329 nm for TiC and 0.4165 nm for VC), making V atoms easily replace Ti atoms in the lattice. The formation of (Ti,V)C enhances the strength and hardness of both the steel matrix and the composites [37].

### 3.3. Chemical Composition of Matrix in Architecture Composites

**Figure 12:**
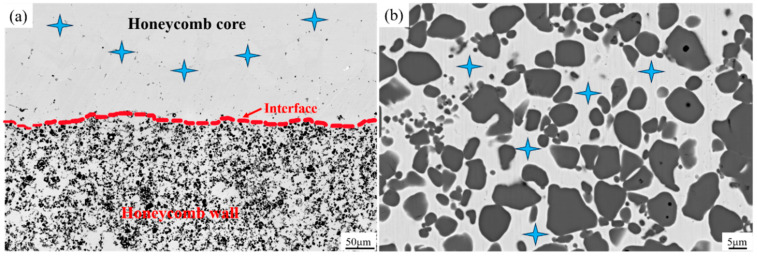
Element distribution in the matrix of the composite contained 50 vol.% composite regions and 50 vol.% TiCp: (**a**) in matrix region; (**b**) in the composite region.

The distribution of elements in the matrix of the composite region was characterized by EPMA. The results are shown in Figure 12 and Table 4, where each value represents the average of the five points examined in Figure 12. The blue elements in Figure 12 indicate the positions where EPMA measurements were taken. According to the results, the content of Cr in the matrix region is approximately 5.5%, and C content is 0.8%. In the composite region, the content of Cr is 6.8% and C content is 1.0%. Compared to the matrix region, the content of Cr and C in the composite region increases slightly. This is because, to adjust the volume fraction of TiCp, certain amounts of Cr-Fe powder and Ni-Fe powder were added during the preparation of the preform, which increased the content of Cr and C and introduced 1.2% Ni. Therefore, despite the high content of C, the content of other elements being close to the standard content of H13 steel shown in Table 1, the introduction of the Ni element promotes interface bonding and enhances the mechanical properties of the composites [38].

### 3.4. Mechanical Properties of Architecture Composites

#### 3.4.1. Hardness of Materials

Table 5 shows the hardness of the composite region in the TiCp/H13 steel architecture composites. The Rockwell C (HRC) scale is commonly used in metallurgy and engineering to measure the hardness of materials such as hardened steels, cast iron, and alloys. In this study, Rockwell hardness is converted from Vickers hardness. The hardness of the matrix region is 53.2 HRC. For the same TiCp content, the hardness of the composite region does not fluctuate greatly with changes in the volume fraction of the composite region. However, for the same composite-region volume fraction, with the TiCp content in the composite region increasing from 35 vol.% to 65 vol.%, the hardness of the composite region of the architecture composites gradually increases, with the highest hardness reaching 66.3 HRC, which is 1.2 times higher than that of the steel matrix.

#### 3.4.2. Compression Properties of Materials

**Figure 13:**
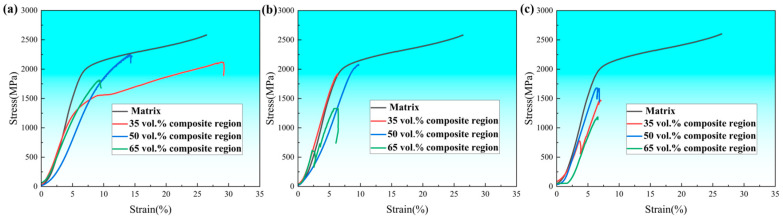
Stress–strain curves of composites with different composite-region fractions: (**a**) 35 vol.% TiCp; (**b**) 50 vol.% TiCp; (**c**) 65 vol.% TiCp.

Figure 13 shows the stress–strain curves of composites with the same TiC content (35 vol.% of TiC, 50 vol.% of TiC, and 65 vol.% of TiC) in different composite regions. As can be seen from the figure, when both the TiCp content (35 vol.%) and composite-region volume fraction (35 vol.%) are low, the honeycomb composite exhibits higher fracture strength and plasticity. However, when either the TiCp content or the composite-region volume fraction is high, the compressive strength and plasticity of the honeycomb composite significantly decrease.

Table 6 and Table 7 present the compressive strength and fracture strain values of the honeycomb-architecture composites under different composite-region volume fractions and TiCp content, respectively. For comparison, the mechanical properties of the H13 steel matrix were also tested, showing a strength of 2578.7 MPa and a corresponding strain of 30.9%.

(1) Effect of the volume fraction of composite region on the strength and plasticity of composites with the same TiCp content

As can be seen from Table 6 and Table 7, for the same TiCp content, the compressive strength of the honeycomb-architecture composites first increases and then decreases with the increase in the volume fraction of the composite region, and the highest strength is achieved at 50 vol.% of the composite region, followed by 35 vol.% of the composite region, and the lowest is 65 vol.% of the composite region. Under the same TiCp content, the average difference between the highest and the lowest strength is 574.9 MPa, so the influence factor of the composite-region fraction on the strength is −38.3 MPa/%. Meanwhile, the fracture strain gradually decreases with the increase in the composite-region fraction. The average difference between the highest and the lowest fracture strain is 7.6%, so the influence factor of composite-region volume fraction on the plasticity of the composites is −0.25%/%.

Compared to the H13 steel matrix, the maximum strength (2288.1 MPa) and plasticity (25.9%) of the architecture composites are lower, in which the strength is reduced by 11.3%, and the fracture strain is reduced by 16.2%.

(2) Effect of TiCp content on the strength and plasticity of composites with the same composite-region fraction

As shown in Table 6 and Table 7, as the increases of TiCp content (35 vol.%, 50 vol.% and 65 vol.%) with the same composite-region fraction, the compressive strength and plasticity of the architecture composites gradually decrease. The average difference between the highest strength (35 vol.% of TiCp) and the lowest strength (65 vol.% TiCp) is 643.2 MPa, so the influence factor of TiCp content on compressive strength is −21.4 MPa/%. The average difference between the highest fracture strain (35 vol.% of TiCp) and the lowest fracture strain (65 vol.% of TiCp) is 10.3%, so the influence factor on plasticity is −0.34%/%.

When comparing the influence factors of the composite region and TiCp content on the strength and plasticity of the architecture composites, the volume fraction of the composite region has a greater effect on strength, while TiCp content has a more significant effect on the plasticity of the architecture composite.

**Figure 14:**
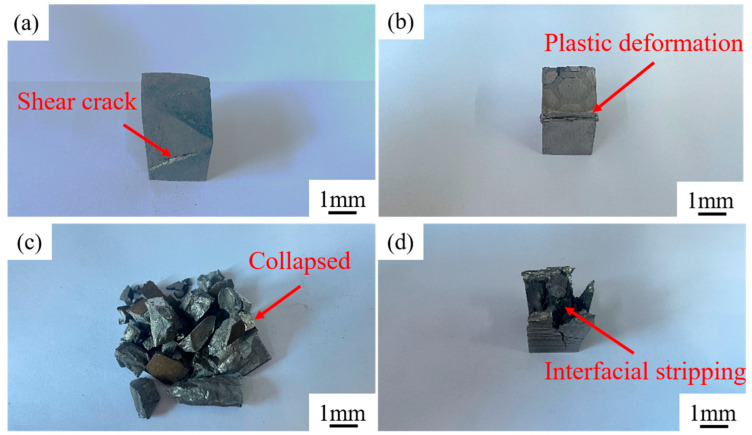
Typical macroscopic morphology of samples after compression: (**a**) matrix; (**b**) composite with 35 vol.% composite region; (**c**) composite with 50 vol.% composite region; (**d**) composite with 65 vol.% composite region.

Figure 14 shows the typical macroscopic morphology of the samples after compression tests. In Figure 14a, for H13 steel, the crack propagates at an angle of 45° along the compression direction [39]. Figure 14b shows the compression sample with 35 vol.% of the composite region, where no obvious cracks appear on the outside of the sample, although obvious plastic deformation occurs. Figure 14c shows the compression sample with 50 vol.% of the composite region, where the composite has collapsed. Figure 14d shows the compression sample with 65 vol.% of the composite region. The cracking occurred at the interface between the composite region and the matrix region, and the composite also collapsed.

**Figure 15:**
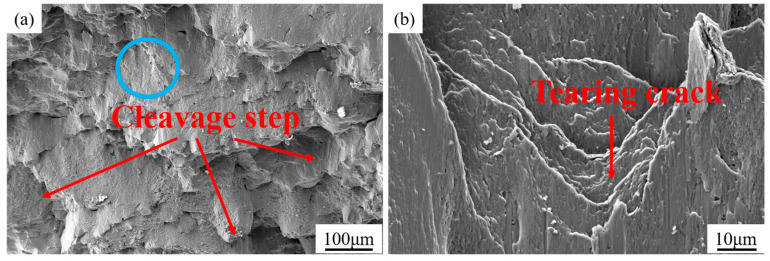
Fracture morphology of matrix after compression: (**a**) fracture morphology at low magnification; (**b**) fracture morphology at high magnification.

Figure 15 shows the fracture microstructure of the matrix steel after compression. In Figure 15a, the fracture surface exhibits an obvious multi-layered step-like structure, with cracks propagating along the dissociation plane, forming a series of parallel cleavage steps at varying heights. Figure 15b provides an enlarged view of the blue region in Figure 15a, highlighting the presence of clear tear patterns.

**Figure 16:**
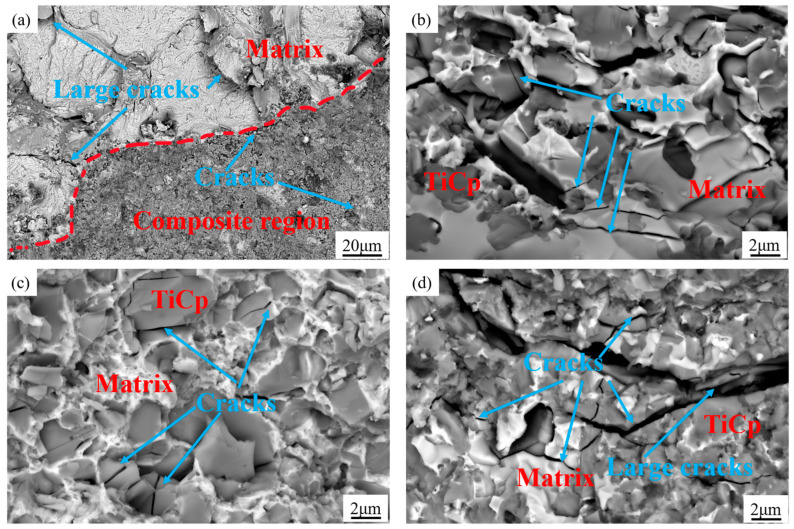
Fracture morphology of composites: (**a**) typical interface between composite and matrix region in 50 vol.% TiCp composites; (**b**) crack propagation in the composite region of 35 vol.% TiC composites; (**c**) crack propagation in the composite region of 50 vol.% TiC composites; (**d**) crack propagation in the composite region of 65 vol.% TiC composites.

Figure 16 shows the fracture morphology of architecture composites with different TiCp content and a 50 vol.% composite-region fraction. Figure 16a shows the microscopic images of the interface between the composite region and the matrix region of 50 vol.% of the TiCp composite. Only a small portion of the crack initiation occurs at the interface, indicating that the interface bonding strength is high. The white matrix area displays obvious river-like patterns and tear patterns, which are characteristics of cleavage fracture or intergranular fracture. However, the fracture surface in the composite region is relatively flat, which indicates that the composite region undergoes a brittle fracture. Figure 16b–d show the compressive fracture microstructure of the composites containing 35 vol.% of TiCp, 50 vol.% of TiCp, and 65 vol.% of TiCp, respectively. When the TiCp content is relatively low, most cracks mainly occur in the H13 steel matrix, accompanied by energy release and material failure during crack propagation.

## 4. Discussion

### 4.1. Comparison of Compression Performance

In this study, honeycomb-architecture composites of H13 steel reinforced by fine TiCp were successfully fabricated, and their compressive strength and fracture strain were significantly improved compared to common high-chromium cast-iron (HCCI)-based honeycomb-architecture composites reinforced by coarse ZTAp. Zhang et al. [27] prepared a ZTAp/HCCI honeycomb-architecture composite using a casting infiltration process, and the results showed that the optimal compressive strength of the composite was 1286.14 ± 15.97 MPa, and the fracture strain was 8.8%. In comparison, the honeycomb composite with 50 vol.% of the composite region and 35 vol.% of TiCp exhibits a strength of 2288.1 MPa and a fracture strain of 13.2%. Therefore, the compressive strength is increased by 1.78 times, and the fracture strain is increased by 1.5 times. Zeng et al. [40] also prepared a ZTAp/HCCI honeycomb composite by a casting infiltration process, incorporating active powder to improve interface bonding. The highest compression strength reaches 1229.62 ± 39.01 MPa, with a fracture strain of 6.32%, when the volume fraction of the composite region is 55%. In comparison, the maximum strength and strain of the 50 vol.% TiC composite in this study are increased by 1.65 times and 1.4 times, respectively. Therefore, through such measurements as select fine (10 μm) TiCp as the reinforcement and H13 steel as the matrix, the strength and plasticity of ceramic-reinforced steel matrix honeycomb composite were significantly enhanced compared to common ZTAp/HCCI composites.

### 4.2. Morphology of Composites with Honeycomb Architecture

#### 4.2.1. Macroscopic Morphology of Sample After Compression

As illustrated in Figure 14, with the increase in the composite region of honeycomb-architecture composite, the honeycomb-architecture composites are more prone to collapse. Therefore, when designing and applying such composites, the influence of the volume fraction of the composite region on crack growth and failure mode should be considered comprehensively, and the appropriate size should be selected to meet specific engineering requirements.

#### 4.2.2. Fracture Morphology of Compressed Samples

Combined with Figure 15 and Figure 16, the fracture of the H13 steel matrix is characterized by a brittle failure mode dominated by cleavage fracture, with a small amount of ductile fracture [41]. In contrast, the fracture of the composite follows a “composite mode fracture”, where internal cracking (brittle behavior) of TiCp and the plastic deformation of the matrix occur simultaneously. Moreover, as the TiCp content increases, cracks mainly form within the TiCp due to its brittleness. Meanwhile, only a few cracks occurred at the TiCp/steel interface, indicating that the TiCp/steel interface is well combined [42].

### 4.3. Formation and Propagation of Internal Cracks in Composites with Honeycomb Architecture

**Figure 17:**
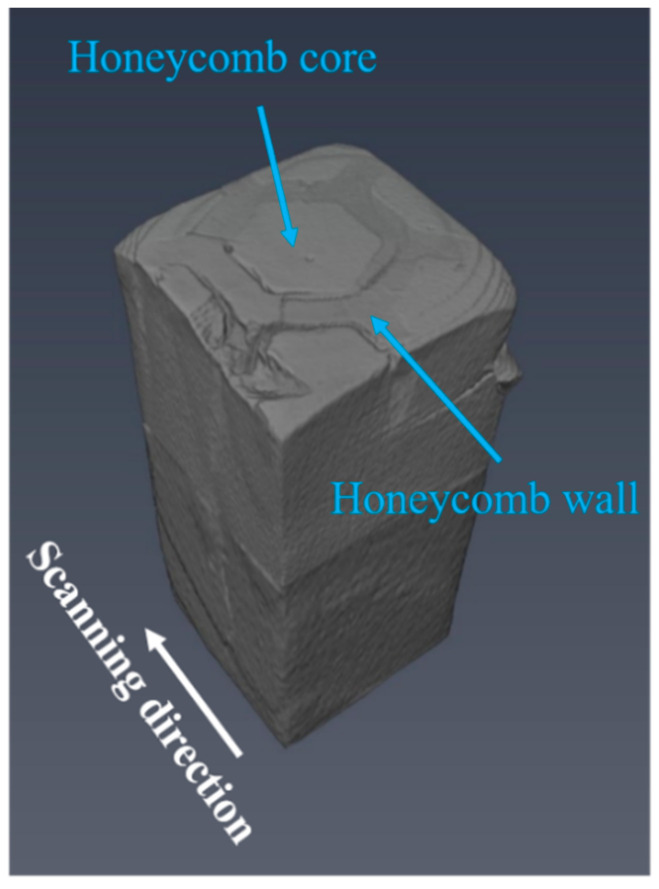
Three-dimensional reconstruction of a compressed sample of composites with 35 vol.% composite region.

Figure 17 shows the reconstructed three-dimensional morphology of compressive specimens of composites from CT tests. The top surface of the figure represents a cell with honeycomb architecture. During the compression process, irregular cracks and shape distortion occur, and the matrix region partially collapses. The top region shows more cracks, indicating the initial failure point. The middle and bottom still maintain integrity but begin transferring stress to the subsiding area at the top. Therefore, honeycomb architecture may exhibit better energy-absorption characteristics during compression, which is one of the advantages of its wide application in high-stress compression structures [43]. Under compressive stress, deformation and collapse occur in the honeycomb wall (composite region) under the action of compressive stress, and the honeycomb core (matrix region) is stripped from the composite region. With the increase in compressive stress, longitudinal interface cracks are connected, and the transverse cracks extend into the composite region, and then the cracks are connected, resulting in the failure of the composites.

**Figure 18:**
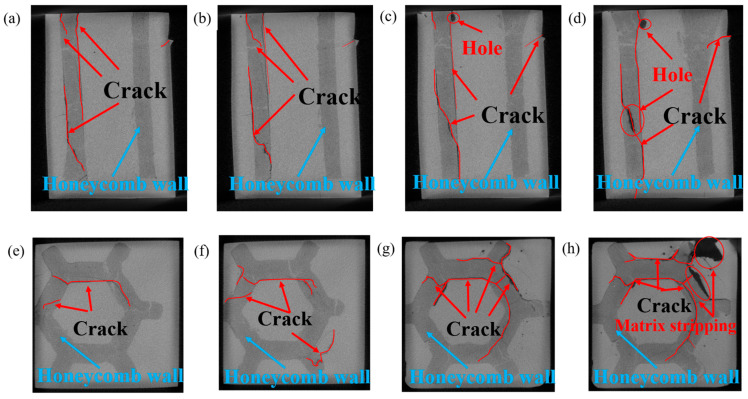
CT images of internal crack growth of composite compressed sample in 35 vol.% composite region: (**a**) the stage of internal crack initiation; (**b**) the stage of internal crack growth; (**c**) the stage of cavity formation; (**d**) the stage of crack penetration; (**e**) initiation stage of top crack; (**f**) the stage of top crack propagation; (**g**) the stage of top crack forming networking; (**h**) the stage of top matrix stripping.

Figure 18 shows the CT images of internal crack growth of the compressed sample with 35 vol.% of the composite region. Figure 18a shows the stage of internal crack initiation. Most of the cracks spread along the vertical direction, which is caused by the shear force between the composite region and the matrix region during compression. Figure 18b shows the internal crack-propagation stage, where the crack length is significantly prolonged. With the progress of compression, the crack still propagates along the direction of the maximum principal stress. Figure 18c shows the stage of cavity formation. The crack length further extends to the composite region. Holes appear at the top, indicating that the local matrix has been stripped or fallen off. The local stress in the composite region is greater, and there is a small deformation. Figure 18d shows the crack penetration stage, in which the number of cracks increases significantly, and the pores become relatively larger. The cracks pass through the matrix from the boundary of the composite region to the entire honeycomb-architecture composite, resulting in a significant decrease in the strength of the honeycomb wall. Figure 18e shows the initiation stage of the top crack. The crack preferentially appears in the interior of the composite region and then spreads and concentrates at the interface between the composite region and the matrix region. The local crack has passed through part of the composite region. Figure 18f shows the crack-propagation stage at the top, where the crack gradually penetrates the composite region and extends to the external matrix, resulting in stress redistribution from the composite region to the matrix region. Figure 18g shows the networking stage of top cracks. The cracks spread along the boundary of the composite region and simultaneously must often change directions. Comparing the honeycomb core (matrix region), the cracks expand to the honeycomb wall (composite region) more easily, indicating that the honeycomb architecture is conducive to improving the plasticity of the material. Figure 18h shows the peeling stage of the top matrix. Cracks penetrate the honeycomb structure, and the matrix shows an obvious peeling phenomenon, resulting in large black holes, and the stress can no longer be effectively transferred, causing the architecture material to fail.

### 4.4. Failure Behavior Comparison Between ZTAp/HCCI and TiCp/H13 Steel of Architecture Composites

The first is the difference in the crack source. According to the analyses above, in the fracture behavior of the TiCp/H13 composites, some TiCps in the steel matrix composites crack during the compression process, but generally, there is only one crack in each TiCp (Figure 15). In contrast, in the ZTAp/HCCI composite, much larger coarse ZTA particles are broken during the compression process, resulting in a large number of cracks. These ZTAps fall off and form more holes. Moreover, many carbides in the high-chromium cast-iron matrix are also prone to cracking [40]. According to GB/T 8263–2010, the tensile strength of HCCI is ≥ 600 MPa, and the impact toughness is ≥4 J/cm^2^. However, according to ASTM A681, the tensile strength of H13 steel is up to 1960 MPa, with an impact toughness of 16 J/cm^2^. Therefore, there are much fewer crack sources for TiCp/H13 steel architecture composites than ZTAp/HCCI architecture composites.

The second is the difference in crack-propagation behavior. In the TiCp/H13 steel composites, cracks preferentially spread along the interface between the composite region and the matrix region and then pass through the interior of the composite region, but they are less likely to spread into the steel matrix. However, in ZTAp/HCCI composites, cracks are first generated at the sharp corners of ZTAp due to the weak bonding between ZTA and iron matrix and the stress concentration effect there; then, many ZTA particles break, fall off, and leave holes, which easily leads to crack propagation [27,44]. Therefore, cracks in the TiCp/H13 steel composites propagate less often than in the ZTAp/HCCI composites.

In summary, the TiCp/H13 steel honeycomb-architecture composites fabricated in this study are superior to the conventional ZTAp/HCCI composites in terms of crack-initiation probability and crack-propagation path, which greatly improves the comprehensive mechanical properties of the honeycomb-architecture composites.

## 5. Conclusions

The TiCp/H13 steel matrix honeycomb composite was successfully prepared by the squeeze-infiltration technique. The effects of TiCp content and the volume fraction of composite region on the compressive properties were studied, and the fracture modes and cracking behaviors of the composite were analyzed. The main conclusions of this study are summarized as follows:

(1) Under the same TiCp content, the compressive strength of honeycomb-architecture composites first increases and then decreases with the increase in composite-region volume fraction, and the highest strength is obtained at 50 vol.% of the composite region. The influence factor of the volume fraction of the composite region on the strength is −38.3 MPa/%. Meanwhile, the fracture strain of the architecture composites gradually decreases. The influence factor of composite-region volume fraction on plasticity is −0.25%/%.
(2) Under the same composite-region fraction, with the increase in TiCp content (35 vol.%, 50 vol.%, and 65 vol.%), the compressive strength and plasticity of the composite both gradually decrease. The influence factor of TiCp content on the strength is −21.4 MPa/%, and its influence factor on plasticity is −0.34%/%.
(3) The maximum compressive strength (2288.1 MPa) is obtained for the architecture composite with a 50 vol.% of the composite region and 35 vol.% of TiCp, and the highest plasticity (25.9%) is obtained for the architecture composite with a 35 vol.% of the composite region and 35 vol.% of TiCp. Compared to traditional ZTA/HCCI composite, its strength and plasticity are significantly improved.
(4) The fracture mode of the architecture composite shows a mixed-mode fracture, i.e., the internal cracking (brittle behavior) of TiCp and the plastic deformation of the matrix work together. The cracks of TiCp/H13 steel honeycomb composite preferentially spread along the interface between the composite region and the matrix region and then through the interior of the composite region.
(5) Compared to common ZTA/iron honeycomb-architecture composites, the TiCp/H13 steel matrix honeycomb-architecture composites use fine particles of TiC instead of coarse particles of ZTA, which greatly reduces the crack initiation and propagation and improves the wettability of ceramic particles and steel matrix. At the same time, H13 steel is used instead of HCCI, which improves the strength and plasticity of the matrix and reduces the cracking of the steel matrix. Therefore, the comprehensive mechanical properties of honeycomb-architecture composites are greatly improved.

## Figures and Tables

**Table 1:** Chemical composition of H13 steel (wt.%).

Element	C	Si	Mn	Cr	Mo	V	P	S	Fe
H13 (wt.%)	0.4	1.0	0.5	5.2	1.5	1.0	≤0.03	≤0.03	89.9

**Table 2:** Parameters of honeycomb-architecture samples.

Volume Fraction of Composite Region (vol.%)	Thickness of Honeycomb Wall (mm)	Sample Size (mm)
35	2.8	20 × 20 × 30
50	3.5	20 × 20 × 30
65	5.2	20 × 20 × 30

**Table 3:** Composite parameters.

	Sample Number	1^#^	2^#^	3^#^	4^#^	5^#^	6^#^	7^#^	8^#^	9^#^
Parameters										
TiCp content (vol.%)		35	35	35	50	50	50	65	65	65
Volume fraction of composite region (vol.%)		35	50	65	35	50	65	35	50	65

# means the sample number.

**Table 4:** Element content in the matrix of composite contained 50 vol.% composite region and 50 vol.% TiCp.

Element	Matrix Region (wt.%)	Matrix in Composite Region (wt.%)
C	0.8	1.0
Cr	5.5	6.8
Mo	1.8	1.8
Si	1.3	1.3
V	0.7	1.0
Ni	0.0	1.2
Fe	89.9	86.9

**Table 5:** Hardness of composite region of architecture composites (HRC).

	35 vol.% Composite Region	Standard Deviation	50 vol.% Composite Region	Standard Deviation	65 vol.% Composite Region	Standard Deviation
35 vol.% TiCp	61.1	0.4	61.5	0.2	62.1	0.5
50 vol.% TiCp	63.1	0.2	63.5	0.4	62.9	0.3
65 vol.% TiCp	66.0	0.1	65.2	0.6	66.3	0.2

**Table 6:** Compressive strength of composites with honeycomb architecture (MPa).

	35 vol.% Composite Region	50 vol.% Composite Region	65 vol.% Composite Region
35 vol.% TiCp	2170.9	2288.1	1809.2
50 vol.% TiCp	1814.8	2032.1	1284.4
65 vol.% TiCp	1500.9	1667.9	1169.8

**Table 7:** Fracture strains of composites with honeycomb architecture (%).

	35 vol.% Composite Region	50 vol.% Composite Region	65 vol.% Composite Region
35 vol.% TiCp	25.9	13.2	9.7
50 vol.% TiCp	9.3	8.9	5.3
65 vol.% TiCp	7.5	5.5	4.8

## Data Availability

The original contributions presented in this study are included in the article. Further inquiries can be directed to the corresponding author.
